# Single-cell transcriptome analysis reveals stem cell-like subsets in the progression of Waldenström’s macroglobulinemia

**DOI:** 10.1186/s40164-023-00382-6

**Published:** 2023-02-17

**Authors:** Yu Qiu, Xiao-shuang Wang, Yuan Yao, Yan-min Si, Xue-zhu Wang, Ming-nan Jia, Dao-bin Zhou, Jia Yu, Xin-xin Cao, Jian Li

**Affiliations:** 1 Department of Hematology, Peking Union Medical College Hospital, Chinese Academy of Medical Sciences and Peking Union Medical College, No. 1 Shuaifuyuan, Beijing, 100730 People’s Republic of China; 2 Department of Hematology, China–Japan Friendship Hospital, Beijing, China; 3 State Key Laboratory of Medical Molecular Biology, Department of Biochemistry and Molecular Biology, Institute of Basic Medical Sciences, Chinese Academy of Medical Sciences and Peking Union Medical College, Beijing, China; 4 State Key Laboratory of Complex Severe and Rare Diseases, Peking Union Medical College Hospital, Beijing, China

## Abstract

Waldenström’s macroglobulinemia (WM) is an uncommon lymphoproliferative disorder, and the precise cellular landscape and the mechanisms of progression from IgM monoclonal gammopathy of undetermined significance (MGUS) to WM remain unclear. We performed single-cell RNA sequencing of CD19 + and CD19-CD38 + cells from healthy donors, IgM MGUS and WM patients. We found that samples from IgM MGUS and WM patients were composed of fewer early B-cell subsets and more T cells and NK cells than those from healthy controls. Compared with those of IgM MGUS patients, mature B cells of WM patients showed upregulation of HES1, GADD45B, NEAT1, DUSP22, RGS1, RGS16, and PIM1. We also identified a subpopulation of CD3 + CD19 + cells in IgM MGUS and WM patients, and trajectory analysis suggested that this subpopulation might be a stem cell-like subset. Further targeted gene sequencing of CD3 + CD19 + and CD3-CD19 + cells proved that MYD88 might be the early events in tumorigenesis according to variant allele fraction analysis. Additional subclonal hits such as CXCR4 and MAP2K1 mutations could be acquired during tumor progression. CXCL signaling, CCL signaling, IL2 signaling and TGFβ signaling pathways were involved in communication between CD3 + CD19 + cells and other immune cells. Our findings reveal the composition of CD38 + immune microenvironment together with B cells and plasma cells in IgM MGUS and WM patients, and provide comprehensive insights into mechanisms of progression from IgM MGUS to WM. The rare CD3 + CD19 + cells might be cells with “stemness” feature.

**Supplementary Information:**

The online version contains supplementary material available at 10.1186/s40164-023-00382-6.

To the editor,

Waldenström’s macroglobulinemia (WM) is an uncommon lymphoproliferative disorder characterized by monoclonal immunoglobulin M protein production and bone marrow infiltration by lymphoplasmacstetytoid cells [1]. It remains an incurable disease. IgM monoclonal gammopathy of undetermined significance (MGUS) is the premalignant condition and is associated with the risk of developing WM [2]. *MYD88*^*L265P*^ is a recurrent mutation found in more than 90% of WM patients, and this genetic event is detectable in at least half of IgM MGUS patients [3]. However, the precise cellular landscape and the mechanisms of progression from IgM MGUS to WM remain unclear. Single-cell RNA sequencing (scRNA-seq) provides us a powerful approach to explore tumor heterogeneity and identify rare malignant cells [4]. Anti-CD38 monoclonal antibodies have been introduced into the therapeutic arsenal for plasma cell diseases [5]. Therefore, we analyzed CD38 + immune microenvironment together with B cells and plasma cells to illustrate the first cellular landscape of IgM MGUS and WM in single cell resolution.

We isolated bone marrow CD19 + and CD19-CD38 + cells from WM patients(n = 3), IgM MGUS patients(n = 3) and healthy donors(n = 3) and employed the 10 × Genomics platform to perform single-cell transcriptomic sequencing. After quality control, our dataset contained a total of 73,024 cells. We used uniform manifold approximation and projection (UMAP) to visualize the cell superclusters and identified clusters including B cells (CD19, MS4A1, and CD79A), plasma cells (SDC1, CD38, and PRDM1), CD3 + CD20 + CD19 + cells, T cells (CD3D, CD8A, and CD4) and NK cells (NCAM1, GNLY, and NKG7) based on the expression of canonical lineage markers and cluster-specific markers (Fig. 1A and Additional file 2: Figure S1A, B). We found that healthy donors had the highest percentage of B cells (72.7% in healthy donors, 19.0% in IgM MGUS patients, 60.5% in WM patients; p < 0.001) and that IgM MGUS and WM patients had more NK and T cells than healthy controls (0.06% and 17.7%, respectively, in healthy donors; 24.1% and 20.5% in IgM MGUS patients; 8.5% and 24.0% in WM patients; p < 0.001) (Fig. 1B). We also noticed that CD3 + CD20 + (CD19 +) cells were present in both IgM MGUS and WM patients (Additional file 2: Figure S1C–E).

**Fig. 1:**
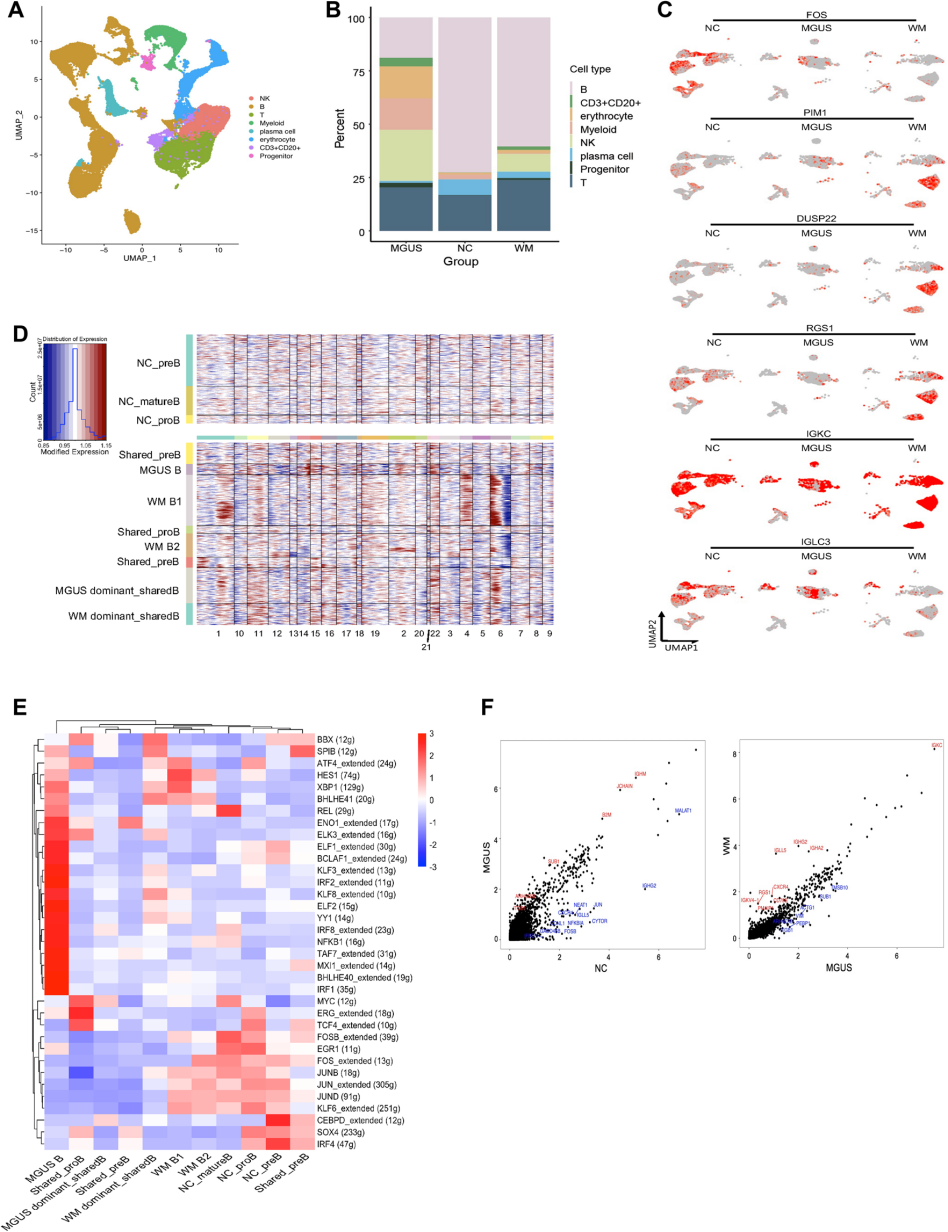
Atlas and Transcriptomic profiling in patients with WM and IgM MGUS. **A** UMAP plot of merged single-cell transcriptomes. Cells are colored according to cell type. **B** Cell proportion of each sub-cluster in three groups. **C** Expression of DEGs of mature B cells and immunoglobin genes in patients or healthy donors. **D** Heatmap showing CNV levels of clusters originated from different samples. Red represents high CNV level and blue represents low CNV level. Horizontal axis represents distribution of genomic regions. **E** SCENIC analysis estimates transcription factors regulating indicated clusters. **F** Scatter plots showing significantly DEGs of plasma cells across the three groups

First, we investigated the cell clusters correlated with WM pathology including B cells and plasma cells. We identified pre-B cells, pro-B cells and mature B cells based on the progenitor cell marker CD34, pre-BCR genes IGLL1 and VPREB1, and B-lymphoid lineage surface markers MME, CD38, FCER2 and CD27. We compared the B-cell gene expression profiles after removing the early B cells (Additional file 2: Figure S1F). Compared with those of IgM MGUS patients, mature B cells of WM patients showed upregulation of HES1, GADD45B, NEAT1, DUSP22, RGS1, RGS16, and PIM1 (Fig. 1C and Additional file 3: Table S1). Light chain restriction and higher copy number variants (CNV) levels were found in B cells of WM patients (Fig. 1C, D). SCENIC analysis showed that FOS, FOSB, EGR1 and JUNB were downregulated in B cells from WM and IgM MGUS patients (Fig. 1E). REL, a proto-oncogene promoting the survival and proliferation of B lymphocytes, was downregulated in B cells of WM patients, and IRF8 and ELF1 were upregulated in B cells of IgM MGUS patients (Fig. 1E). Nevertheless, plasma cells of WM patients, IgM MGUS patients and healthy donors were colocalized in one cluster, which might indicate their similar gene expression patterns. The significant differentially expressed genes (DEGs) across the groups are highlighted in scatter plots (Fig. 1F) and showed in Additional file 4: Table S2. In particular, we noticed that the expression levels of part of genes decrease in IgM MGUS patients and then rise again when the disease progresses to WM. We assumed that the regulation might be correlated with the progression from IgM MGUS to WM.

Next, we further determined the characteristics of the rare CD3 + CD19 + cells. Trajectory analysis revealed that CD3 + CD20 + (CD19 +) cells and pre-B cells were located at the same end of the branched structure and were then directed toward mature B cell and plasma cell fates, respectively (Fig. 2A). This inferred developmental trajectory suggested that CD3 + CD20 + (CD19 +) cells may be tumor stem cell-like subset. Using flow cytometry, we validated that CD3 was expressed in a fraction of CD19 + cells (mean: 3.69%, range: 0.28%-15.10%) of bone marrow samples from 8 WM patients (Fig. 2B, Additional file 2: Figure S2A and Additional file 5: Table S3). We compared the gene expression of CD3 + CD20 + CD19 + cells between WM patients and IgM MGUS patients (Additional file 6: Table S4). Genes including IGKC, JCHAIN, AREG, RGS1, IGHM were significantly upregulated in CD3 + CD20 + CD19 + cells of WM patients. In addition, targeted gene sequencing (TGS) was performed on CD3 + CD19 + and CD3-CD19 + cells from 6 WM patients (Additional file 7: Table S5). MYD88 were proved to be the most frequently mutated gene in this cell subset (Fig. 2C). And we inferred that MYD88 mutation might be the early events in tumorigenesis by variant allele fraction (VAF) analysis (Fig. 2D, E). Additional subclonal hits, such as CXCR4 and MAP2K1 mutations, could be acquired during tumor progression.

**Fig. 2:**
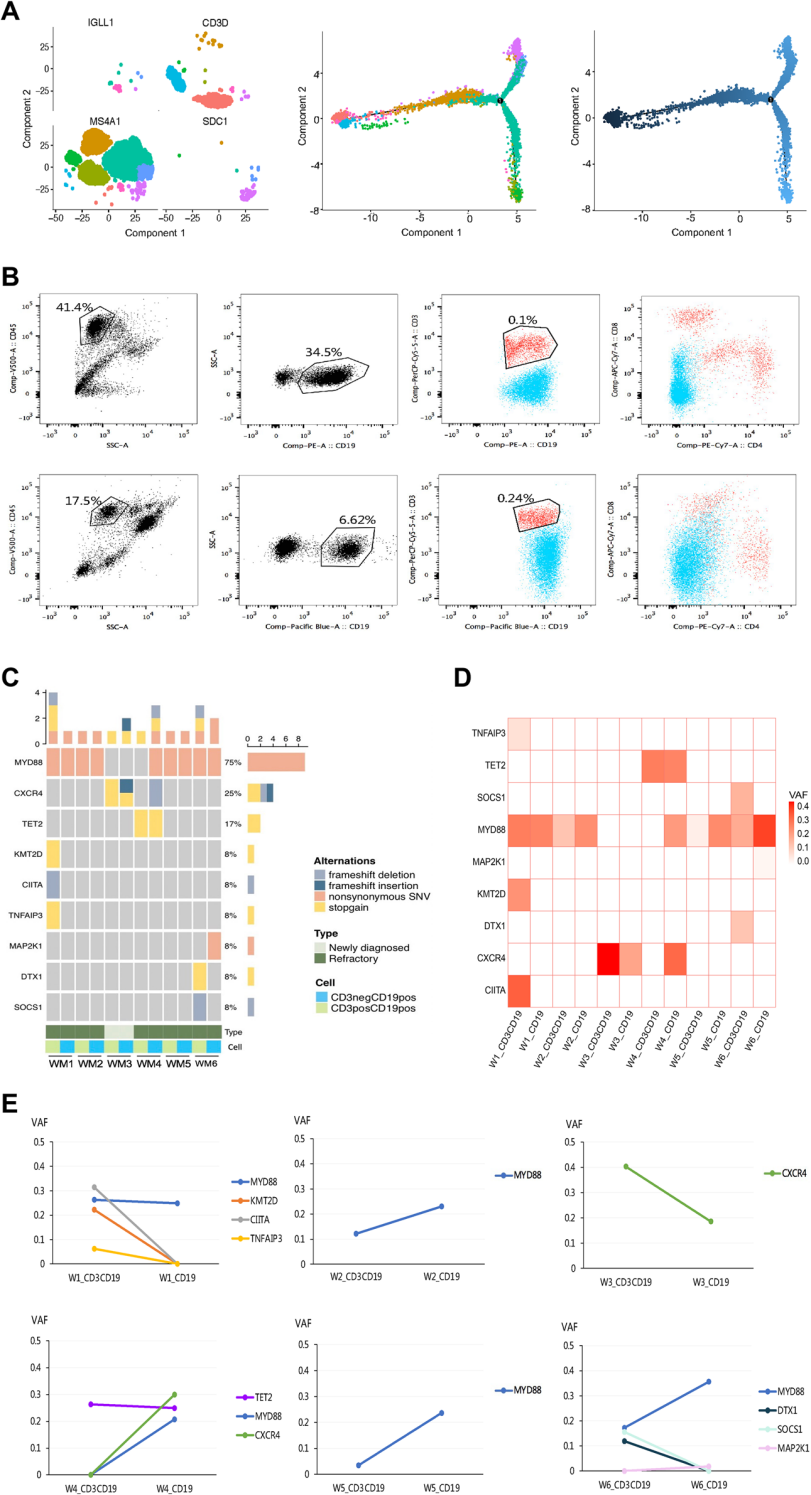
Stem cell-like subset in patients with WM and its mutational landscape. **A** Pseudo-time analysis of B cells, B cells with aberrant T cell markers, plasma cells inferred by Monocle 2. Expression of classical markers shows clusters identification: CD3 + CD20 + cells and preB cells (IGLL1 +) are located in the “root” (pseudo-time 0) and B cells (MS4A1 +) and plasma cells (SDC1 +) are located in the branch. **B** Flow cytometric analysis showing the population of B cells with aberrant T cell markers in two WM patients. CD3 + CD19 + cells were labeled with red color. And expression of CD4 and CD8 in CD3 + CD19 + cells were analyzed. **C** Mutant genes identified by target region sequencing in CD3 + CD19 + cells and CD3-CD19 + cells of 6 patients with WM. Mutation types and mutation rate of each gene were displayed in the right side. **D** Heatmap showing VAF distribution of nine mutated genes. **E** Dynamics of VAF across CD3 + CD19 + cells and CD3-CD19 + cells

We observed higher proportions of T lymphocytes and NK cells in WM patients; thus, *CellChat* was employed to explore the cell–cell communication between immune cells and CD3 + CD20 + (CD19 +) cells in WM patients. We observed that the CXCR4-CXCL12 ligand-receptor pair was enriched in the interaction between CD3 + CD20 + cells and B cells (Additional file 2: Figure. S2B, C). The CCL signaling pathway were highly expressed in CD8 + T cell/NK-cell—CD3 + CD20 + cell interactions (Additional file 2: Figure. S2B, C). In addition, IL2 signaling, as well as TGF β signaling, were also involved in communication networks between CD3 + CD20 + cells and T/NK cells (Additional file 2: Figure. S2B, C).

In conclusion, our study provides comprehensive insights into mechanisms of progression from IgM MGUS to WM. We identified the rare CD3 + CD19 + cell subpopulation in WM patients. It will be interesting to explore novel therapeutic strategies targeting rare potential cells with “stemness” in future.

## Supplementary Information

**Additional file 1: Table S1.** Demographics of patients. **Table S2.** Summary and quality of sequencing. **Table S3.** Gene list of targeted gene sequencing panel

**Additional file 2: Figure S1. **Cellular landscape of patients with WM and IgM MGUS. A. UMAP plot of merged single-cell transcriptomes. Cells are colored according to sample origin. B. Dot plot of feature genes expression in each cluster. C. UMAP plot showing clusters identified in WM patients. D. UMAP plot showing clusters identified in healthy donors. E. UMAP plot showing clusters identified in IgM MGUS patients. F. UMAP plot of B cell sub-clusters in patients and healthy donors. **Figure S2. **Identification of CD3+CD19+ cells and cell-cell communication. A. Flow cytometric analysis showing the population of CD3+CD19+ cells in six WM patients. B. Cell-cell communication inferred by Cellchat. C.Relative contribution of each ligand-receptor pair.

**Additional file 3.** Differentially expressed genes of mature B cells.

**Additional file 4.** Differentially expressed genes of plasma cells.

**Additional file 5: Table S3.** Percentage of CD3+CD19+ cells in 8 WM samples.

**Additional file 6.** Differentially expressed genes of CD3+CD19+ cells between WM and IgM MGUS patients.

**Additional file 7: Table S5.** Mutation in the 6 WM patients

## Data Availability

ScRNA-seq data has been deposited to National Genomics Data Center with accession number HRA002419 [6, 7]. Code is available on request to the corresponding author.

## Abbreviations

WM: Waldenström’s macroglobulinemia
MGUS: Monoclonal gammopathy of undetermined significance
scRNA-seq: Single-cell RNA sequencing
UMAP: Uniform manifold approximation and projection
TGS: Targeted gene sequencing
DEGs: Differentially expressed genes
CNV: Copy number variants
VAF: Variant allele fraction
