# Supplementary material for: Single-cell transcriptome analysis reveals stem cell-like subsets in the progression of Waldenström’s macroglobulinemia
Source: Exp Hematol Oncol. 2023 Feb 17;12:18. doi: 10.1186/s40164-023-00382-6 (PMC9936698; doi:10.1186/s40164-023-00382-6)
Supplement: Supplementary file 1 — Additional file 1: Table S1. Demographics of patients. Table S2. Summary and quality of sequencing. Table S3. Gene list of targeted gene sequencing panel [file 40164_2023_382_MOESM1_ESM.docx]

**Methods**
**Human samples**

A total of 9 bone marrow specimens from patients with newly diagnosed symptomatic WM patients (n=3), patients with newly diagnosed IgM MGUS (n=3) and healthy donors (n=3) were used for 10x Genomics single-cell analysis. Diagnosis of WM was made according to the criteria of the Second International Workshop on WM(1). IgM MGUS is defined as the presence of IgM monoclonal protein less than 3 g/dL, bone marrow lymphoplasmacytoid cells infiltration less than 10% and absence of end-organ damage related to monoclonal protein(2, 3). Bone marrow samples from one of the abovementioned WM patients and 7 additional WM patients were used for subsequent flow cytometric analysis. Sorted cells from 6 of these 8 WM patients were subjected to next-generation sequencing. The demographic characteristics of the patients are summarized in Supplementary Table 1. Written Informed consent was obtained from all patients, and the study was approved by the Peking Union Medical College Hospital Ethics Committee. The current study was performed in accordance with the ethical standards of the 1964 Declaration of Helsinki and its later amendments.

**Cell isolation, library construction and sequencing**

Bone marrow aspirates (20 ml) were collected in ethylenediaminetetraacetic acid (EDTA)-containing tubes and immediately transported on ice. Bone marrow aspirates were diluted 1:1 in ice-cold fluorescence-activated cell sorting (FACS) buffer (2 mM EDTA and 2% FBS in PBS), and mononuclear cells were obtained by density centrifugation as previously described(4). Bone marrow mononuclear cells were carefully washed with ice-cold FACS buffer and then stained with antibodies against CD38 (BD, clone HIT2, cat. no. 555460) and CD19 (BD, clone HIB19, cat. no. 555412); both CD19+ and CD19-CD38+ cells were then collected using an MA900 Flow Cell Sorter (SONY). Cell viability was examined by staining with 0.4% trypan blue, and then the sorted cells were resuspended in PBS containing 0.04% bovine serum albumin (BSA). The concentration of the single-cell suspension was adjusted to 1000 cells/μl. Cells were loaded to achieve recovery of approximately 10000 cells. Library construction was performed according to the Chromium Single Cell 3’ Reagent Kit v2 User Guide. Single-cell libraries were subjected to 150 bp paired-end sequencing on the Illumina HiSeq platform.

**Data processing**

Cell Ranger (version 3.0.2, 10x Genomics) was employed to process the raw sequencing output data. FASTQ files were aligned to the human reference genome GRCh38, filtered and then used to generate a gene-barcode matrix using the Cell Ranger count pipeline. The quality of the sequencing reads is listed in Supplementary Table 2. We used the R package Seurat (v3.1) to perform quality control and downstream analysis of the output data(5). Low-quality cells (cell with<200 or >2500 genes/cell, nUMI<1000, mitochondrial genes>5%, and ribosomal UMIs>20%) were removed. We merged all datasets and performed normalization using the *LogNormalize* function. The 2000 most highly variable genes were selected using the *FindVariableGenes* function and used to perform dimensionality reduction. Principal components were determined using the *PCElbowPlot* function. We visualized clusters using uniform manifold approximation and projection (UMAP). Subclustering of B-cell clusters was performed in the same workflow. Cell cycle analysis was performed using the *CellCycleScoring* function, which performs calculations based on cell cycle markers.

**Differential gene expression analysis**
Cell identities were assigned based on the expression levels of the known marker genes and cluster-specific markers. We used the *FindMarkers* function to identify differentially expressed genes among clusters. Significant differentially expressed genes were identified as those with an average logFC ≥1 and a P value < 0.05. We applied the R package *clusterProfiler* and Metascape to perform Gene Ontology (GO) term and KEGG pathway enrichment analyses, and Gene set enrichment analysis (GSEA)(6, 7).

**Trajectory analysis**

The Monocle2 package was used to construct the potential lineage trajectories among cell clusters, including B cells, plasma cells and CD3+CD20+ cells. Dimensionality reduction was performed using DDRTree algorithm in the *reduceDimension* function. Pro-B cells and pre-B cells were assigned as the root state when using the *orderCell* function.

**SCENIC analysis and CNV estimation**

SCENIC analysis was conducted using the SCENIC package (version 1.2.4) in R to explore the single-cell regulatory network(8). The expression matrix of B cells from Seurat was used as input for SCENIC analysis. We used the cisTarget human databases hg19-500bp-upstream and hg19-tss-centered-10kb to identify and score the motifs. The “top5perTarget” method was used in identification of regulons. The InferCNV package was used to detect CNVs in B cells. Four B subclusters from healthy donors were used as the controls.

**Cell–cell communication analysis**

Cell-cell communication in WM patients was evaluated using CellChat(9). Metadata for major cell clusters were used as input for CellChat. We conducted this analysis via a previous workflow and used default parameters. We quantified ligand-receptor pairs among different cell types and visualized them using a circle plot.

**Flow cytometry**

Bone marrow mononuclear cells of 2 patients with newly diagnosed WM and 6 patients with relapsed or refractory WM were suspended in ice-cold FACS buffer. Cells were analyzed by flow cytometry using sets of antibodies against ckappa (FITC)/clambda (PE)/CD3 (PerCP)/CD19 (PECy7)/CD38 (APC)/CD45 (V500)/CD20 (APC Cy7) or CD20 (FITC)/CD19 (PE)/CD3 (PerCP)/CD38 (APC)/CD4 (PE Cy7)/CD7 (BV421)/CD8 (APC Cy7)/CD45 (V500). The ratio of CD19+ CD3+ cells was analyzed by flow cytometry. Data acquisition was performed in a FACSCanto II flow cytometer (BD Biosciences) using FACSDiva software (BD Biosciences).

**Next-generation sequencing**

A targeted gene sequencing (TGS) panel including 69 genes that were related to lymphoproliferative diseases was designed. The details of the 69 targeted genes are summarized in Supplementary Table 3. Genomic DNA from CD19+ CD3- and CD19+ CD3+ cells was prepared and sequenced. Paired-end reads were aligned to the hg19 reference human genome with Burrows–Wheeler Aligner (BWA) software. SAMtools was applied for somatic single nucleotide variation (SNV) calling. Then, mutations were filtered and annotated using ANNOVAR.

**Statistics**

All statistical analyses were performed in R (version 3.6.0). We used the default Wilcoxon rank-sum test implemented in Seurat.

**References**

1. Owen RG, Treon SP, Al-Katib A, Fonseca R, Greipp PR, McMaster ML, et al. Clinicopathological definition of Waldenstrom's macroglobulinemia: consensus panel recommendations from the Second International Workshop on Waldenstrom's Macroglobulinemia. Seminars in oncology. 2003;30(2):110-5.

2. Mailankody S, Landgren O. Monoclonal gammopathy of undetermined significance and Waldenström's macroglobulinemia. Best Practice & Research Clinical Haematology. 2016;29(2):187-93.

3. Mouhieddine TH, Weeks LD, Ghobrial IM. Monoclonal gammopathy of undetermined significance. Blood. 2019;133(23):2484-94.

4. Ledergor G, Weiner A, Zada M, Wang SY, Cohen YC, Gatt ME, et al. Single cell dissection of plasma cell heterogeneity in symptomatic and asymptomatic myeloma. Nature medicine. 2018;24(12):1867-76.

5. Satija R, Farrell JA, Gennert D, Schier AF, Regev A. Spatial reconstruction of single-cell gene expression data. Nature biotechnology. 2015;33(5):495-502.

6. Yu G, Wang LG, Han Y, He QY. clusterProfiler: an R package for comparing biological themes among gene clusters. Omics : a journal of integrative biology. 2012;16(5):284-7.

7. Zhou Y, Zhou B, Pache L, Chang M, Khodabakhshi AH, Tanaseichuk O, et al. Metascape provides a biologist-oriented resource for the analysis of systems-level datasets. Nature communications. 2019;10(1):1523.

8. Aibar S, González-Blas CB, Moerman T, Huynh-Thu VA, Imrichova H, Hulselmans G, et al. SCENIC: single-cell regulatory network inference and clustering. Nature methods. 2017;14(11):1083-6.

9. Jin S, Guerrero-Juarez CF, Zhang L, Chang I, Ramos R, Kuan CH, et al. Inference and analysis of cell-cell communication using CellChat. Nature communications. 2021;12(1):1088.

Table S1. Demographics of patients

| Sample | Age | Sex | Sample |
| --- | --- | --- | --- |
| WM1 | 62 | male | Flow cytometry, Target region sequencing |
| WM2 | 49 | male | Flow cytometry, Target region sequencing |
| WM3 | 72 | female | Flow cytometry, Target region sequencing |
| WM4 | 67 | male | Flow cytometry, Target region sequencing |
| WM5 | 69 | male | Flow cytometry, Target region sequencing |
| WM6 | 62 | male | Flow cytometry, Target region sequencing |
| WM7 | 53 | male | Flow cytometry |
| WM8 | 58 | male | ScRNA, Flow cytometry |
| WM9 | 73 | male | ScRNA |
| WM10 | 75 | male | ScRNA |
| IgM MGUS1 | 60 | male | ScRNA |
| IgM MGUS2 | 41 | male | ScRNA |
| IgM MGUS3 | 80 | male | ScRNA |
| Healthy donor1 | 69 | male | ScRNA |
| Healthy donor2 | 72 | female | ScRNA |
| Healthy donor3 | 24 | female | ScRNA |

Table S2. Summary and quality of sequencing

| Sample | Number of cells sequenced | Median genes per cell | Sequencing Saturation, % | Fraction reads, % | Reads mapped to exonic regions, % |
| --- | --- | --- | --- | --- | --- |
| WM8 | 6,716 | 933 | 88.0 | 75.4 | 68.3 |
| WM9 | 7,854 | 934 | 88.8 | 95.5 | 72.0 |
| WM10 | 5,735 | 1,301 | 88.4 | 96.2 | 63.9 |
| IgM MGUS1 | 3,276 | 1,023 | 89.8 | 62.2 | 69.9 |
| IgM MGUS2 | 5,778 | 1,047 | 90.6 | 91.2 | 67.0 |
| IgM MGUS3 | 26,598 | 880 | 67.4 | 71.8 | 65.2 |
| Healthy donor1 | 6,913 | 1,044 | 84.2 | 96.6 | 73.0 |
| Healthy donor2 | 7,144 | 1,152 | 84.4 | 88.1 | 65.5 |
| Healthy donor3 | 8,423 | 1,003 | 86.0 | 94.3 | 62.7 |

Table S3. Gene list of targeted gene sequencing panel

| Gene list | CytoBand |
| --- | --- |
| APC | 5q22.2 |
| ARID1A | 1p36.11 |
| ARID1B | 6q25.3 |
| ARID2 | 12q12 |
| ATM | 11q22.3 |
| B2M | 15q21.1 |
| BCL2 | 18q21.33 |
| BCORL1 | Xq26.1 |
| BIRC3 | 11q22.2 |
| BRAF | 7q34 |
| CARD11 | 7p22.2 |
| CCND1 | 11q13.3 |
| CCND3 | 6p21.1 |
| CD28 | 2q33.2 |
| CD58 | 1p13.1 |
| CD79B | 17q23.3 |
| CHD8 | 14q11.2 |
| CIITA | 16p13.13 |
| CREBBP | 16p13.3 |
| CXCR4 | 2q22.1 |
| DNMT3A | 2p23.3 |
| DTX | 12q24.13 |
| EBF1 | 5q33.3 |
| EP300 | 22q13.2 |
| EZH2 | 7q36.1 |
| FOXO1 | 13q14.11 |
| GNA13 | 17q24.1 |
| ID3 | 1p36.12 |
| IDH2 | 15q26.1 |
| IRF4 | 6p25.3 |
| IRF8 | 16q24.1 |
| ITPKB | 1q42.12 |
| JAK1 | 1p31.3 |
| JAK3 | 19p13.11 |
| KDM6A | Xp11.3 |
| KMT2A | 11q23.3 |
| KMT2C | 7q36.1 |
| KMT2D | 12q13.12 |
| MAP2K1 | 15q22.31 |
| MEF2B | 19p13.11 |
| MFHAS1 | 8p23.1 |
| MYC | 8q24.21 |
| MYD88 | 3p22.2 |
| NF1 | 17q11.2 |
| NOTCH1 | 9q34.3 |
| NOTCH2 | 1p12 |
| PIM1 | 6p21.2 |
| PRDM1 | 6q21 |
| PTEN | 10q23.31 |
| PTPN1 | 20q13.13 |
| RHOA | 3p21.31 |
| SF3B1 | 2q33.1 |
| SOCS1 | 16p13.13 |
| SPEN | 1p36.13 |
| STAT3 | 17q21.2 |
| STAT5B | 17q21.2 |
| STAT6 | 12q13.3 |
| TCF3 | 19p13.3 |
| TET2 | 4q24 |
| TNFAIP3 | 6q23.3 |
| TP53 | 17p13.1 |
| TRAF3 | 14q32.32 |
| XPO1 | 2p15 |
| ZAP70 | 2q11.2 |
| TNFRSF14 | 1p36.32 |
| IL2RG | Xq13.1 |
| FYN | 6q21 |
| CD79A | 19q13.2 |
| ADAM3A | 8p11.22 |
