# Supplementary material for: Single-cell transcriptome analysis reveals stem cell-like subsets in the progression of Waldenström’s macroglobulinemia
Source: Exp Hematol Oncol. 2023 Feb 17;12:18. doi: 10.1186/s40164-023-00382-6 (PMC9936698; doi:10.1186/s40164-023-00382-6)
Supplement: Supplementary file 2 — Additional file 2: Figure S1. Cellular landscape of patients with WM and IgM MGUS. A. UMAP plot of merged single-cell transcriptomes. Cells are colored according to sample origin. B. Dot plot of feature genes expression in each cluster. C. UMAP plot showing clusters identified in WM patients. D. UMAP plot showing clusters identified in healthy donors. E. UMAP plot showing clusters identified in IgM MGUS patients. F. UMAP plot of B cell sub-clusters in patients and healthy donors. Figure S2. Identification of CD3+CD19+ cells and cell-cell communication. A. Flow cytometric analysis showing the population of CD3+CD19+ cells in six WM patients. B. Cell-cell communication inferred by Cellchat. C.Relative contribution of each ligand-receptor pair. [file 40164_2023_382_MOESM2_ESM.docx]

**Figure S1. Cellular landscape of patients with WM and IgM MGUS**

A. UMAP plot of merged single-cell transcriptomes. Cells are colored according to sample origin. B. Dot plot of feature genes expression in each cluster. C. UMAP plot showing clusters identified in WM patients. D. UMAP plot showing clusters identified in healthy donors. E. UMAP plot showing clusters identified in IgM MGUS patients. F. UMAP plot of B cell sub-clusters in patients and healthy donors.

**Figure S2. Identification of CD3+CD19+ cells and cell-cell communication**

A. Flow cytometric analysis showing the population of CD3+CD19+ cells in six WM patients. B. Cell-cell communication inferred by Cellchat. C.Relative contribution of each ligand-receptor pair.
